# Myélome multiple à IgD mimant un myélome à chaines légères libres et compliqué de déficit en facteur X: à propos d'un cas

**DOI:** 10.11604/pamj.2022.41.338.31755

**Published:** 2022-04-27

**Authors:** Najat Lasri, Fatimazahra Lahlimi, Mohammed Ilias Tazi, Sophie Rigaudeau

**Affiliations:** 1 Service d´Hématologie Clinique et de Greffe de Moelle, Centre Hospitalier Universitaire Mohammed VI, Faculté de Médecine et de Pharmacie, Université Cadi Ayyad, Marrakech, Maroc,; 2 Service d´Hématologie Clinique, Centre Hospitalier de Versailles André Mignot, Faculté de Médecine Xavier Bichat, Université Paris Diderot, Paris, France

**Keywords:** IgD, chaînes légères, troubles d´hémostase, cas clinique, IgD, light chains, haemostasis disorders, case report

## Abstract

Le myélome à IgD est une hémopathie extrêmement rare dont la présentation clinique est sévère. Il peut prêter à confusion avec le myélome non sécrétant ou à chaines légères libres. Nous rapportons le cas d´une patiente âgée de 72 ans qui s´est présentée pour des douleurs osseuses et ecchymoses diffuses dans un contexte d´altération de l´état général. Le bilan réalisé a montré une gammapathie monoclonale associée à une insuffisance rénale aigüe sévère et à un TP bas à 48% avec un déficit en facteur X. Le bilan étiologique a confirmé le diagnostic de myélome à IgD lambda stade IIIb selon Durie et Salmon, score “International Staging System” (ISS) à III de cytogénétique défavorable. L´évolution a été favorable après traitement par inhibiteur de protéasome, anti-CD 38 et corticothérapie. Le traitement adéquat du myélome à IgD en utilisant les nouvelles approches thérapeutiques et l´autogreffe des cellules souches hématopoïétiques améliore le pronostic.

## Abstract (en)

IgD myeloma is an extremely rare haemopathy with severe clinical presentation. It can be confused with non-secretory or free light chain myeloma. We here report the case of a 72-year old female patient presenting with bone pain and diffuse ecchymosis and deterioration of her general condition. Laboratory tests showed monoclonal gammopathy associated with severe acute renal failure and low total protein (TP) (48%) with factor X deficiency. Etiological assessment confirmed the diagnosis of IgD lambda myeloma stage IIIb, according to Durie and Salmon, International Staging System (ISS) score III unfavorable cytogenetics. Patient’s outcome was favorable after treatment with proteasome inhibitor, anti-CD 38 and corticosteroid therapy. Adequate treatment of IgD myeloma, using new therapeutic approaches and hematopoietic stem cell autotransplantation, can improve the prognosis.

## Introduction

Le myélome multiple à IgD fait partie des gammapathies monoclonales les plus rares. Le diagnostic peut prêter à confusion avec le myélome à chaînes légères libres ou non sécrétant [1]. Il représente moins de 2% des myélomes [2]. La présentation clinique est souvent agressive rendant le pronostic péjoratif dans la plupart des cas [1,2]. Il existe un risque de confusion avec le myélome non sécrétant ou à chaînes légères libres. Les troubles d´hémostase sont expliqués par la séquestration des facteurs de coagulation par les chaines légères libres ou la production des inhibiteurs [2,3]. Les progrès thérapeutiques récents ont permis d´améliorer le devenir des patients ayant un myélome multiple à IgD y compris pour les plus fragiles non éligibles à une intensification thérapeutique [1]. Nous rapportons le cas d´un myélome multiple à IgD lamba diagnostiqué et traité dans le Service d´Hématologie Clinique du Centre Hospitalier de Versailles avec une bonne évolution au décours.

## Patient et observation

**Informations du patient:** une patiente âgée de 72 ans ayant comme antécédent une dyslipidémie, était admise pour des douleurs osseuses évoluant dans un contexte d´altération de l´état général.

**Résultats cliniques:** la patiente avait un *performans status* à 4, un syndrome hémorragique (ecchymose palpébrale droite ainsi qu´au niveau des sites d´injection), une impotence fonctionnelle partielle du membre inférieur droit avec un clinostatisme et des douleurs à la palpation de l´aine et à la mobilisation de la hanche droite. L´examen neurologique était normal.

**Figure 1:**
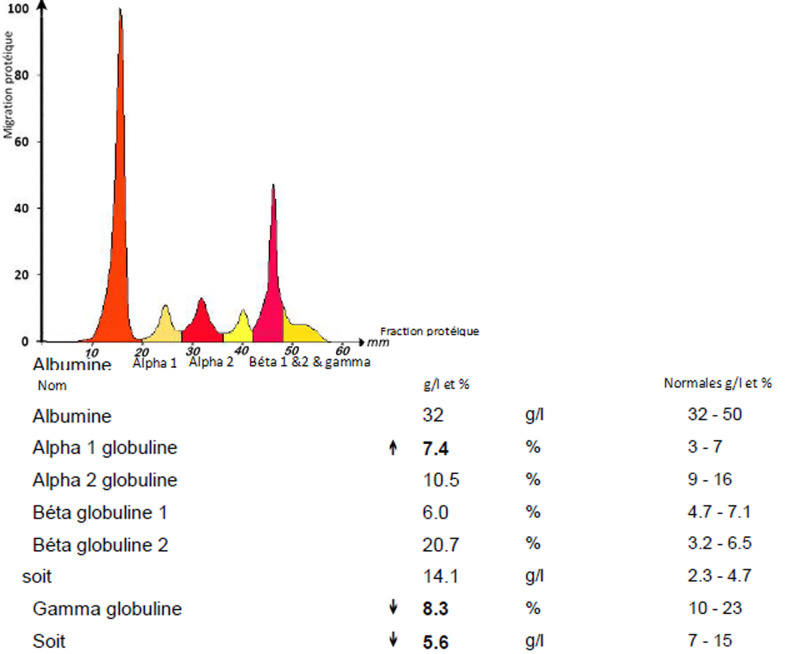
électrophorèse des protéines plasmatiques montrant un pic monoclonal à 14,1 g/l au niveau de la zone des béta-globulines, avec une hypogammaglobulinémie à 5,6 g/l, traduisant la présence de gammapathie monoclonale en rapport avec le myélome diagnostiqué chez notre patiente

**Figure 2:**
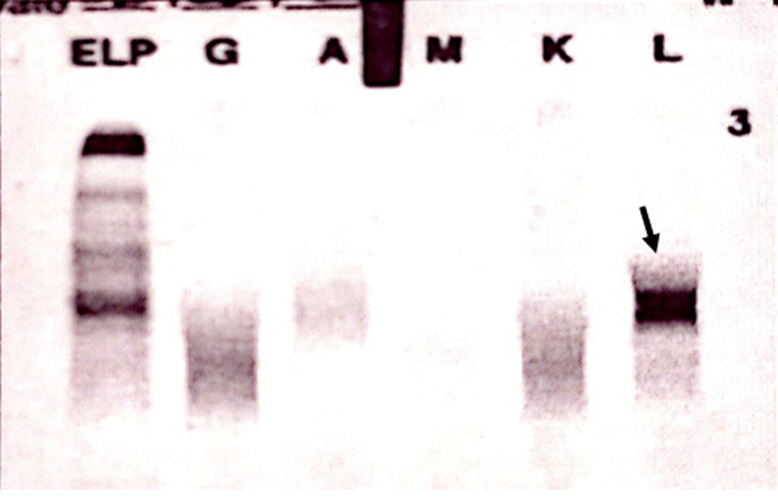
immunofixation des protéines plasmatiques montrant une bande monoclonale d´isotype lambda sans réactivité avec une chaîne lourde α, δ ou µ, permettant d´écarter le myélome multiple à IgA, IgG, et IgM chez notre patiente

**Figure 3:**
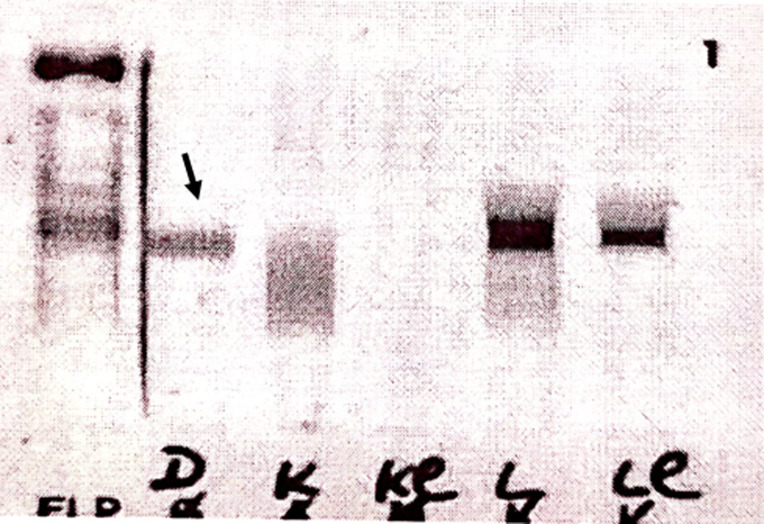
seconde immunofixation des protéines plasmatiques montrant la présence d´une IgD lambda monoclonale associée à la présence de chaînes légères lambda, permettant de poser le diagnostic du myélome multiple à IgD lambda avec excès de production des chaînes légères lambda

**Démarche diagnostique:** le bilan biologique a révélé une anémie normocytaire arégénérative à 9,6 g/dl sans autres anomalies de la numération formule sanguine (NFS), un TP bas à 48% avec un déficit en facteur X sans inhibiteur ni autres anomalies hémostatiques, une insuffisance rénale aigüe (IRA) sévère avec une créatinine à 639 µmol/l (débit de filtration glomérulaire à 6 ml/min/1,73 m^2^) et une hypercalcémie (calcémie corrigée) à 3,19 mmol/l. Le dosage pondéral de l´albumine était à 27 g/l, la béta2-microglobuline à 34 mg/l chez une patiente en IRA soit un ISS (International Staging System) à 3. Les LDH étaient élevés à 1,5 fois la normale, la CRP était à 16 mg/l. L´électrophorèse des protéines sériques a révélé l´existence d´un pic monoclonal migrant dans la zone des bétaglobulines avec des béta-globulines+pic à 14 g/l (Figure 1). L´immunofixation réalisée en utilisant initialement les anti-sérums des immunoglobulines IgG, IgA, IgM a été négative (Figure 2). L´analyse a été complétée par une immunofixation en utilisant l´anti-sérum IgD qui est revenue positive en montrant la présence d´une IgD lambda monoclonale associée à la présence de chaînes légères libres lambda prédominantes (Figure 3). Le dosage des chaînes légères libres (CLL) lambda a été à 26122 mg/l, contre 20,31 mg/l pour les CLL kappa. Nous avons constaté une protéinurie massive à 8.21g/24h de type glomérulaire et tubulaire faite des chaines légères lambda. Il n´y a eu pas d´arguments justifiant la ponction biopsie rénale. La moelle osseuse a été envahie par 34% de plasmocytes dystrophiques. La Fish a retrouvé une amplification partielle 1 q.

**Figure 4:**
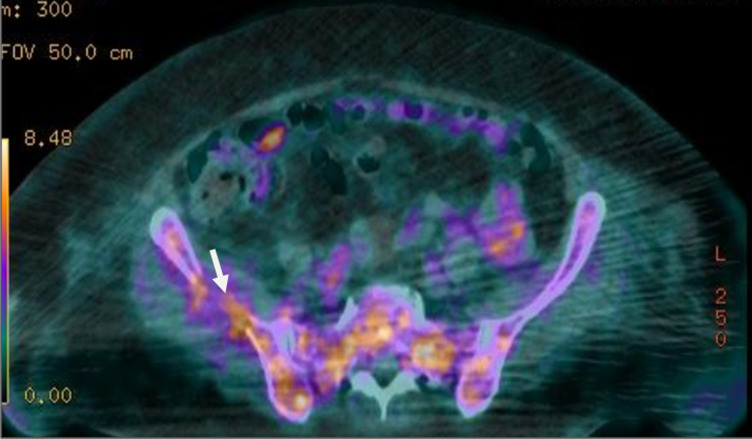
image du PET scanner montrant des lésions hypermétaboliques au niveau du bassin plus marquées au niveau cotyloïdien droit avec fracture de l´acétabulum, en rapport avec l´ostéolyse secondaire au myélome diagnostiqué chez notre patiente

**Figure 5:**
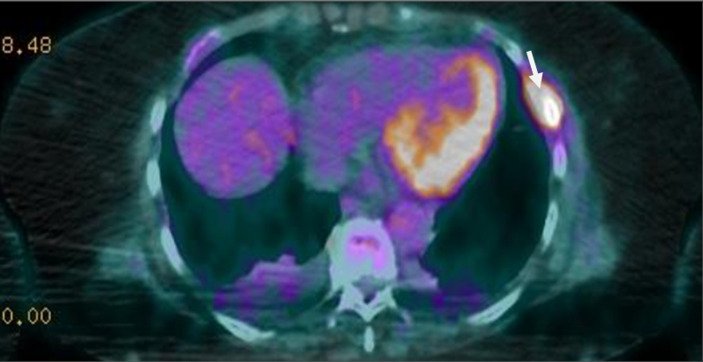
image du PET scanner montrant des lésions hypermétaboliques au niveau costal en rapport avec l´ostéolyse secondaire au myélome diagnostiqué chez notre patiente

**Figure 6:**
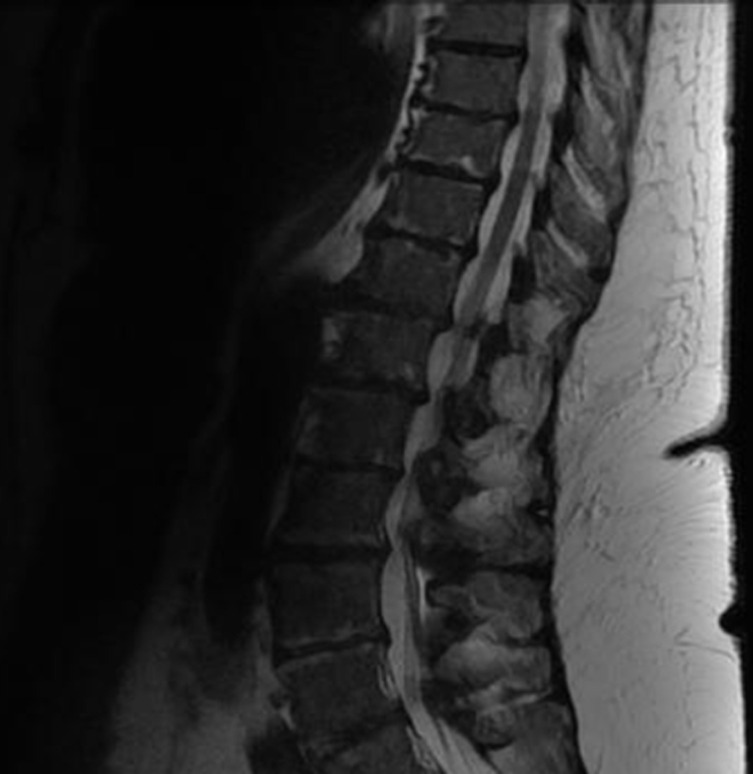
IRM du rachis lombaire montrant une perte complète du signal graisseux des vertèbres sur l´ensemble du rachis visible traduisant un remplacement médullaire diffus en rapport avec le myélome diagnostiqué chez notre patiente

Une l'imagerie par résonance magnétique (IRM) cardiaque, une biopsie des glandes salivaires et de la graisse sous cutanée n´ont pas retrouvé d´arguments en faveur d´une amylose. La tomographie par émission de positons (TEP) a révélé de multiples lésions hypermétaboliques notamment costales et cotyloïdiennes droites avec une fracture de l´acétabulum (Figure 4, Figure 5). L´IRM du rachis lombaire a révélé une perte complète du signal graisseux des vertèbres sur l´ensemble du rachis visible traduisant un remplacement médullaire diffus (Figure 6). Ainsi le diagnostic de myélome à IgD lambda stade IIIb selon Durie et Salmon, score ISS à III de cytogénétique défavorable a été posé.

**Intervention thérapeutique:** la patiente, non éligible à l´autogreffe, a d´abord était traitée par une chimiothérapie de type VCD (Velcade hebdomadaire-cyclophosphamide 1 bolus unique 1000 mg et dexaméthasone 20 mg/jour x 4 jours) vu la présence d´IRA sévère. Au 20^e^ jour de cette chimiothérapie on notait l´amélioration de l´état général de la patiente, de la fonction rénale avec une créatinine à 212 μmol/l et l´obtention de rémission partielle. La patiente a ensuite été traitée par le protocole daratumumab-velcade-dexaméthasone (DVD). Sur le plan osseux, un traitement mensuel par biphosphonates a été débuté. Les fractures du cotyle et de l´os iliaque droit ont été traitées par ostéosynthèse avec cimentoplastie et haubanage.

**Suivi et résultats:** aucun incident n´a été noté au cours du traitement en dehors d´un épisode infectieux documenté à l´entérocoque facium et au staphylocoque doré MétiS avec une bonne évolution au décours de l´antibiothérapie à base de daptomycine. Notre patiente a reçu jusque-là quatre cycles de DVD, et est actuellement en très bonne réponse partielle avec des chaînes légères libres lambda à 48 mg/l vs 26122 mg/l. On note une reprise de l´autonomie et de la marche, une correction de l´anémie et une nette amélioration de la fonction rénale avec une créatinine à 100 µmol/l.

## Discussion

Le myélome à IgD est une hémopathie rare [1,2]. Il représente moins de 2% des myélomes, la prédominance est masculine [1]. L´âge moyen est plus bas par rapport aux autres sous types de myélome (entre 54 et 65 ans selon les séries) [1,3]. Il se caractérise par la sévérité de la présentation clinique avec de fréquentes localisations extra-médullaires qui peuvent être atypiques (testicule, foie, rate, rein) [4], d´ostéolyse importante faisant des douleurs osseuses le mode de révélation le plus fréquent. L´insuffisance rénale est fréquente, cette dernière influence négativement le devenir du patient quand elle devient irréversible [1-3,5]. Le pic monoclonal est souvent discret voire absent, car la synthèse des IgD est dix fois inférieure à celles des autres immunoglobulines [6], ce qui fait confondre ce myélome avec un myélome non sécrétant. Dans la série de Gertz *et al*. un pic n´est décrit que dans 0,7% des cas de myélome à IgD [5]. Quand il est détectable, comme dans notre cas, il est le plus souvent dans la zone des béta-globulines. On peut aussi confondre le myélome à IgD avec un myélome à chaînes légères notamment lambda compte tenu de la fréquence du sous type IgD lambda (90%) dont le taux joue un rôle pronostique comme rapporté dans la littérature [1,2,6]. L´immunotypage avec la recherche des anti-corps anti-chaînes delta et epsilon ainsi que le dosage des nouveaux biomarqueurs (N-glycanes) aident au diagnostic devant le risque de confusion avec les myélomes à chaînes légères [7]. Par contre, l´immunotypage ne permettant pas la caractérisation des IgD et des IgE, l´immunofixation en utilisant les antisérums anti-IgD et anti-IgE peut être réalisée devant la mise en évidence des chaînes légères libres [8].

La protéinurie est majoritairement positive. Comme dans notre cas, elle est le plus souvent massive, elle est faite de chaînes légères dans 71% des cas dans la série de Djidjik *et al*. [1]. L´insuffisance rénale est fréquente et rapportée le plus souvent à la néphrotoxicité accrue des chaînes légères lambda en comparaison avec les chaînes kappa [3,9]. Elle peut être aggravée par l´hypercalcémie, c´est ce qu´on note chez notre patiente (DFG à 6 ml/min/m).

Certaines séries rapportent une amylose associée au myélome à IgD avec la présence de dépôts amyloïdes au niveau de la graisse sous cutanée dans 73% des cas, de la biobsie ostéomédullaire dans 47% des cas, et plus rarement au niveau de la peau ou de la rate [5]. Djidjik *et al*. ont retrouvé une localisation cardiaque dans 45% des cas [1]. Les troubles d´hémostase sont expliqués par la séquestration des facteurs de coagulation par les chaînes légères libres ou la production des inhibiteurs [10]. La recherche d´inhibiteur chez notre patiente était négative. La correction du facteur X en parallèle de la baisse des chaînes légères libres après le traitement permet de conclure à l´hypothèse de séquestration. La B2 microglobuline, marqueur de la masse tumoral et pronostique, est souvent élevée, mais l´interprétation est difficile devant la fréquence du contexte d´insuffisance rénale [1-3].

Sur le plan pronostic, la cytogénétique est défavorable dans 33% à 53% des cas [6]. Dans la série rétrospective de Liu *et al*. qui recense 1068 cas de myélome dont 356 cas de myélome multiple à IgD, la translocation (11,14) est la plus fréquente, dans ce sous type d´hémopathie le remaniement du chromosome 1 retrouvé chez notre patiente représente 30,1% des cas dans cette série [2]. La survie médiane liée à cette anomalie chromosomique est estimée à 14,5 mois selon Selene *et al*. [6].

Les progrès thérapeutiques récents ont permis d´améliorer le devenir des patients ayant un myélome multiple, par l´utilisation de triplette ou de quadruplette (inhibiteur de protéasome +/- immunomodulateurs +/- anti-CD 38 et corticothérapie). La survie médiane dans le myélome à IgD est inférieure à celle des autres sous types de myélome [2]. La survie globale est améliorée par l´intensification thérapeutique et l´autogreffe des cellules souches hématopoïétiques chez les sujets éligibles après le traitement d´induction [2].

**Point de vue de la patiente:** au décours d´hospitalisation, l´évolution a été rapidement favorable, la patiente a ressenti une très bonne amélioration après les soins prodigués.

**Consentement éclairé:** nous avons obtenu le consentement éclairé de la patiente pour utiliser les images dans ce rapport de cas.

## Conclusion

Le myélome multiple à IgD est une entité rare. Le risque de confusion avec les myélomes non secrétant ou à chaînes légères libres constitue un piège diagnostique. Le tableau clinique est le plus souvent agressif, toutefois des cas ayant une évolution favorable sont décrits. Comme pour tous les myélomes, le pronostic est amélioré par l´utilisation de nouvelles approches thérapeutiques et à la greffe des cellules souches hématopoïétiques.
